# CAP-LAMP2b–Modified Stem Cells’ Extracellular Vesicles Hybrid with CRISPR-Cas9 Targeting ADAMTS4 to Reverse IL-1β–Induced Aggrecan Loss in Chondrocytes

**DOI:** 10.3390/ijms26199812

**Published:** 2025-10-09

**Authors:** Kun-Chi Wu, Yu-Hsun Chang, Raymond Yuh-Shyan Chiang, Dah-Ching Ding

**Affiliations:** 1 Department of Orthopedics, Hualien Tzu Chi Hospital, Buddhist Tzu Chi Medical Foundation, Tzu Chi University, Hualien 970, Taiwan; drwukunchi@yahoo.com.tw; 2 Department of Pediatrics, Hualien Tzu Chi Hospital, Buddhist Tzu Chi Medical Foundation, Tzu Chi University, Hualien 970, Taiwan; cyh0515@gmail.com; 3 Department of Obstetrics and Gynecology, Hualien Tzu Chi Hospital, Buddhist Tzu Chi Medical Foundation, Tzu Chi University, Hualien 970, Taiwan; raymond880106@gmail.com; 4 Institute of Medical Sciences, Tzu Chi University, Hualien 970, Taiwan

**Keywords:** human umbilical cord mesenchymal stem cells, extracellular vesicles, cartilage repair, inflammation, CAP-LAMP2b engineering, ADAMTS4, regenerative medicine

## Abstract

Extracellular vesicles (EVs) from mesenchymal stem cells hold therapeutic promise for inflammatory and degenerative diseases; however, limited delivery and targeting capabilities hinder their clinical use. In this study, we sought to enhance the anti-inflammatory and chondroprotective effects of EVs through CAP-LAMP2b (chondrocyte affinity peptide fused to an EV membrane protein) engineering and *ADAMTS4* gene editing hybrid vesicle formation. Human umbilical cord MSCs (hUCMSCs) were characterized via morphology, immunophenotyping, and trilineage differentiation. EVs from control and CAP-LAMP2b-transfected hUCMSCs were fused with liposomes carrying CRISPR-Cas9 ADAMTS4 gRNA. DiI-labeled EV uptake was assessed via fluorescence imaging. CAP-LAMP2b was expressed in hUCMSCs and their EVs. EVs exhibited the expected size (~120 nm), morphology, and exosomal markers (CD9, CD63, CD81, HSP70). CAP-modified hybrid EVs significantly enhanced chondrocyte uptake compared to control EVs and liposomes. IL-1β increased ADAMTS4 expression, whereas CAP-LAMP2b-ADAMTS4 EVs, particularly clone SG3, reversed these effects by reducing ADAMTS4 and restoring aggrecan. Western blotting confirmed suppressed ADAMTS4 and elevated aggrecan protein. CAP-LAMP2b-ADAMTS4 EVs, therefore, showed superior uptake and therapeutic efficacy in inflamed chondrocytes, attenuating inflammatory gene expression and preserving matrix integrity. These results support engineered EVs as a promising cell-free approach for cartilage repair and osteoarthritis treatment.

## 1. Introduction

Osteoarthritis (OA) is the most prevalent joint disorder and a leading cause of disability worldwide, with incidence increasing due to aging populations [1]. In 2020, 595 million people worldwide (7.6%, 95% uncertainty interval 6.8–8.4) had osteoarthritis—an increase of 132.2% since 1990—with cases projected to rise by 74.9% for knee, 48.6% for hand, 78.6% for hip, and 95.1% for other sites by 2050 [1]. Once regarded as a cartilage-limited disease, OA is now recognized as a multifactorial disorder involving the entire joint, driven by complex interactions between local and systemic factors [2]. Age and obesity are the principal risk factors, and the disease markedly reduces quality of life in older adults [3]. Current pharmacological treatments are restricted to symptom relief, and no disease-modifying OA drugs have been approved, underscoring the urgent need for novel therapeutics [2].

OA is characterized by the degradation of articular cartilage, particularly the loss of aggrecan, a proteoglycan that imparts load-bearing and compressive resistance properties to cartilage [4]. Aggrecan degradation is mediated by matrix metalloproteinases (MMPs) and aggrecanases, most notably ADAMTS4 and ADAMTS5 [5]. Aggrecanase-driven breakdown represents an early and pivotal event in OA progression, leading to cartilage erosion and joint dysfunction [6]. Given their central role, ADAMTS4 and ADAMTS5 have emerged as promising therapeutic targets [5].

CRISPR/Cas9 genome editing offers a precise and efficient method for targeting disease-associated genes [7]. The system utilizes guide RNA to direct the Cas9 nuclease to specific genomic loci, inducing double-strand breaks that are repaired by cellular mechanisms, thereby enabling gene knockout or modification [8]. CRISPR/Cas9 has been widely applied in biomedical research and is under investigation for treating genetic and degenerative diseases, including arthritis [9]. Targeted silencing of ADAMTS4 using CRISPR/Cas9 may mitigate cartilage degradation and slow the progression of OA, offering a potential disease-modifying therapy [10].

Human umbilical cord-derived mesenchymal stem cells (hUCMSCs) are an attractive therapeutic platform due to their high proliferative capacity, potent immunomodulatory effects, and secretion of extracellular vesicles (EVs) that facilitate tissue repair [11,12]. EVs can be engineered as delivery vehicles for genome-editing tools, providing a minimally invasive and targeted therapeutic approach [11,12].

EVs secreted by hUCMSCs have intrinsic biocompatibility, the capacity to protect cargo from enzymatic degradation, and efficient cellular uptake [11,12]. In our study, we further enhanced these inherent advantages by engineering the lysosome-associated membrane protein 2b (LAMP2b) with a chondrocyte-affinity peptide (CAP) on the EV membrane [13,14]. This hybrid CAP-LAMP2b modification increases EV tropism toward chondrocytes, facilitating selective binding and internalization. By combining this targeting strategy with the encapsulation of ADAMTS4-directed gRNA/CRISPR-Cas9 complexes, we exploit the EV platform to achieve precise and efficient delivery of gene-editing tools to chondrocytes.

In this study, we hypothesize that this strategy would suppress aggrecan degradation, enhance cartilage protection, and offer a novel, less invasive therapeutic option for OA management.

## 2. Results

### 2.1. Characterization of Human Umbilical Cord-Derived Mesenchymal Stem Cells

**Figure 1:**
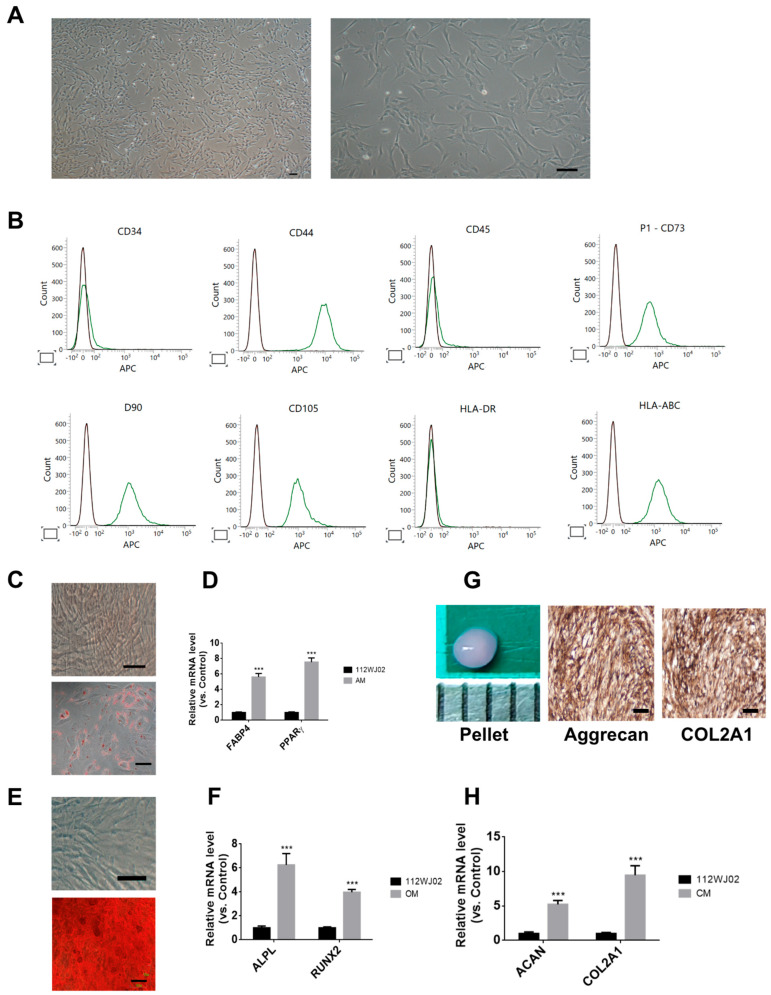
Characterization of human umbilical cord-derived mesenchymal stem cells (hUCMSCs)**.** (**A**) Morphology of cultured hUCMSCs observed under a light microscope at early passages, showing typical fibroblast-like spindle-shaped morphology. Scale bar: 100 µm. (**B**) Flow cytometry analysis of surface markers. hUCMSCs were positive for CD44, CD73, CD90, CD105, and HLA-ABC, and negative for hematopoietic markers CD34, CD45, and HLA-DR, consistent with the MSC phenotype. The green line represents cells stained with specific APC-conjugated antibodies, indicating positive expression of the corresponding surface markers. (**C**) The upper panel showed no staining control. The lower panel showed Oil Red O staining showing lipid droplet accumulation after adipogenic induction. Scale bar = 100 μm. (**D**) Quantitative RT-PCR demonstrating elevated expression of adipogenic markers FABP4 and PPARγ in adipogenic medium (AM) compared to that in control. (**E**) The upper panel showed no staining control. The lower panel showed Alizarin Red S staining showing calcium deposition after osteogenic induction. Scale bar = 100 μm. (**F**) qRT-PCR analysis of osteogenic markers ALPL and RUNX2 expression in osteogenic medium (OM) compared to that in control. (**G**) The left panel shows pellet formation after 21 days of chondrogenesis (Scale bar = 1mm). The middle and right panels showed immunohistochemical staining of chondrogenic pellets showing positive staining for aggrecan and COL2A1. Scale bar = 100 μm. (**H**) qRT-PCR showing increased *ACAN* and *COL2A1* mRNA expression in chondrogenic medium (CM) compared to that in the control. Data are presented as mean ± SEM; *** *p* < 0.001 vs. control.

In this study, hUCMSCs exhibited a typical fibroblast-like morphology under a light microscope (Figure 1A); expressed surface markers consistent with MSC identity, including CD44, CD73, CD90, CD105, and HLA-ABC; and were negative for CD34, CD45, and HLA-DR (Figure 1B). Trilineage differentiation was confirmed: adipogenic differentiation was evidenced via Oil Red O staining and increased FABP4 and PPARγ expression (Figure 1C,D); osteogenic differentiation via Alizarin Red S staining and upregulation of ALPL and RUNX2 (Figure 1E,F); and chondrogenic differentiation via immunohistochemical detection of aggrecan and COL2A1, along with elevated *ACAN* and *COL2A1* mRNA levels (Figure 1G,H). These findings confirm the multipotency and immunophenotype of hUCMSCs.

### 2.2. Detection of FLAG-Tagged CAP-LAMP2b Expression in hUCMSC-Derived Exosomes and Cells

**Figure 2:**
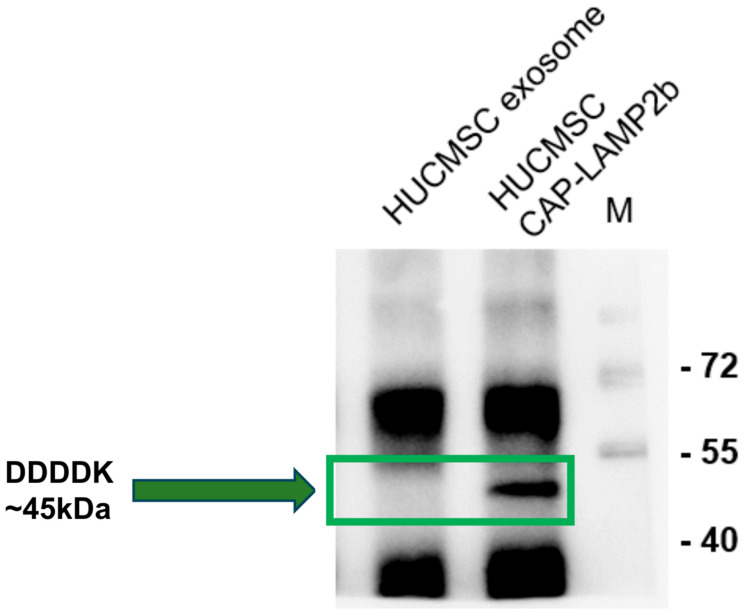
Detection of FLAG-tagged CAP-LAMP2b expression in hUCMSC-derived exosomes and cells. Western blot using anti-DDDDK (FLAG) antibody shows a specific band at ~45 kDa in hUCMSCs transfected with CAP-LAMP2b construct and in the corresponding exosomes, indicating successful expression and packaging of the fusion protein. The ~45 kDa band, highlighted by a green box and arrow, is absent in control hUCMSC exosomes, confirming specificity. Molecular weight markers (M) are shown on the right.

As shown in Figure 2, Western blot using an anti-FLAG (DDDDK) antibody confirmed expression of the CAP-LAMP2b fusion protein in both hUCMSCs and their exosomes, with a specific band at approximately 45 kDa. This band appeared only in CAP-LAMP2b-transfected hUCMSCs and corresponding exosomes, but not in untransfected exosome controls, indicating efficient incorporation of the FLAG-tagged construct into exosomal cargo.

### 2.3. Characterization of hUCMSC-EVs, CAP-LAMP2b-EVs, and CAP-LAMP2b-ADAMTS4 EVs

**Figure 3:**
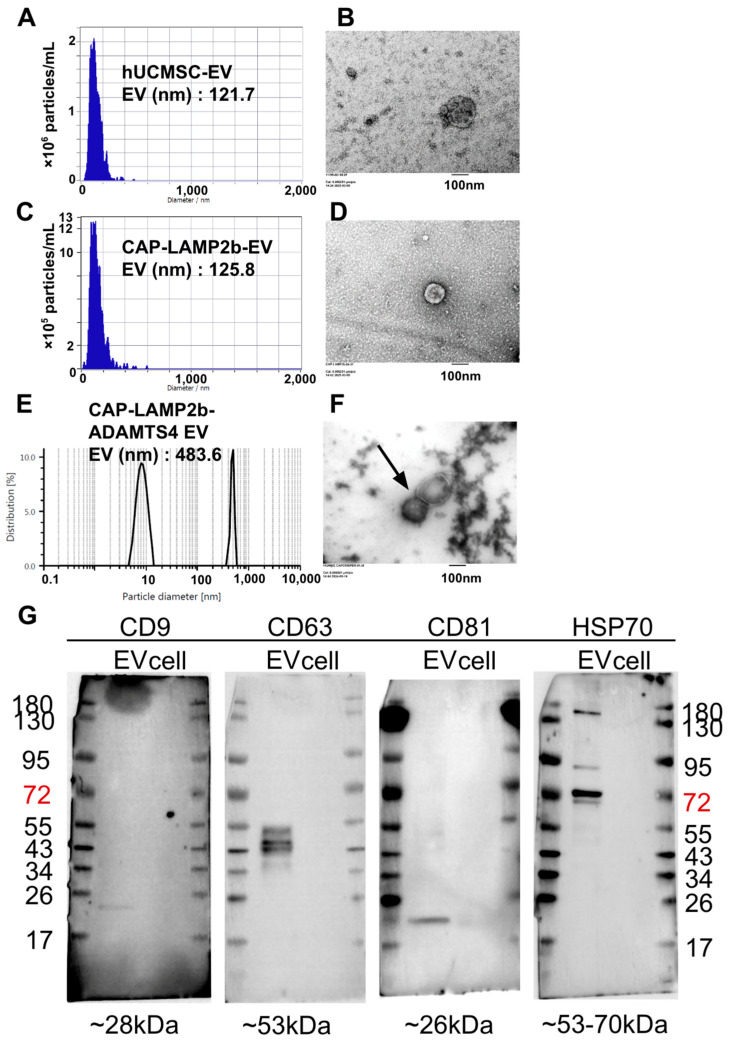
Characterization of extracellular vesicles (EVs) derived from hUCMSC, CAP-LAMP2b-expressing hUCMSCs, and CAP-LAMP2b-ADAMTS4 EVs. (**A**,**C**,**E**) Nanoparticle tracking analysis (NTA) of hUCMSC-EVs, CAP-LAMP2b-EVs, and CAP-LAMP2b-ADAMTS4 EVs showing average particle sizes of 121.7 nm, 125.8 nm, and 483.6 nm, respectively. (**B**,**D**,**F**) Transmission electron microscopy (TEM) images demonstrating the typical cup-shaped morphology of isolated EVs from hUCMSC-EVs. Scale bar = 100 nm. (**B**), CAP-LAMP2b-EVs (**D**), and CAP-LAMP2b-ADAMTS4 EVs (arrow) (**F**). (**G**) Western blot analysis confirming the presence of established EV markers (CD9, CD63, CD81, and HSP70) in both EVs and EV-producing cells. Bands appear at the expected molecular weights, validating EV identity: ~28 kDa (CD9), ~53 kDa (CD63), ~26 kDa (CD81), and ~53–70 kDa (HSP70). The number is molecular weight of the proteins.

As shown in Figure 3, EVs derived from hUCMSCs and CAP-LAMP2b-transfected hUCMSCs, and CAP-LAMP2b-ADAMTS4 EVs, were characterized via NTA, which revealed comparable mean diameters of 121.7 nm, 125.8 nm, and 483.6 nm, respectively (Figure 3A,C,E). TEM confirmed the typical spherical morphology of EVs in both groups (Figure 3B,D,F). Western blot analysis demonstrated the presence of canonical EV markers, including CD9, CD63, CD81, and HSP70, in both EVs (Figure 3G) and their parental cells, validating the successful isolation and identity of the vesicles.

### 2.4. Enhanced Cellular Uptake of CAP-Modified Hybrid EVs

**Figure 4:**
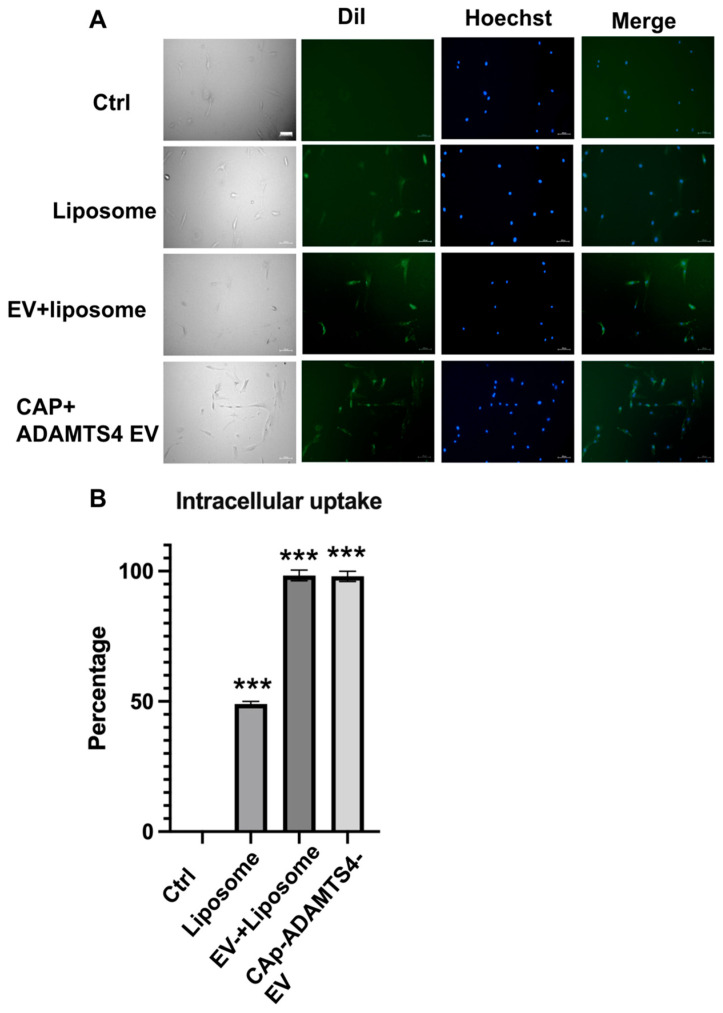
Enhanced cellular uptake of CAP-modified hybrid extracellular vesicles (EVs). (**A**) Fluorescence microscopy images showing intracellular uptake of DiI-labeled liposomes, EV + liposome, and CAP-ADAMTS4 EV by recipient cells (chondrocytes). Hoechst staining (blue) marks nuclei, and DiI signal (green) indicates internalized vesicles. Merged images demonstrate a significantly stronger DiI signal in the EV + liposome and CAP-ADAMTS4-EV groups compared to the liposome and control groups. Scale bar = 100 μm. (**B**) Quantification of intracellular uptake using fluorescence microscopy images. Both the EV + liposome and CAP-ADAMTS4-EV groups exhibited significantly higher uptake efficiency than the liposome controls (*** *p* < 0.001), confirming enhanced delivery and cell internalization by the engineered EVs. Ctrl: control.

As shown in Figure 4, CAP-modified hybrid EVs exhibited enhanced cellular uptake. Fluorescence microscopy revealed stronger DiI signals in cells treated with hUCMSC-EV + liposome and CAP-ADAMTS4 EV compared to liposome-treated or untreated controls, indicating greater internalization. Quantitative analysis confirmed that both hUCMSC-EV + liposome and CAP-ADAMTS4 EV groups displayed significantly higher intracellular uptake (*p* < 0.001) than liposome controls, demonstrating that hUCMSC-EV + liposome and CAP-ADAMTS4 EV effectively enhanced EV delivery to target cells.

### 2.5. Western Blot Analysis of ADAMTS4 Protein Expression Following EV Treatment in IL-1β-Stimulated Chondrocytes

**Figure 5:**
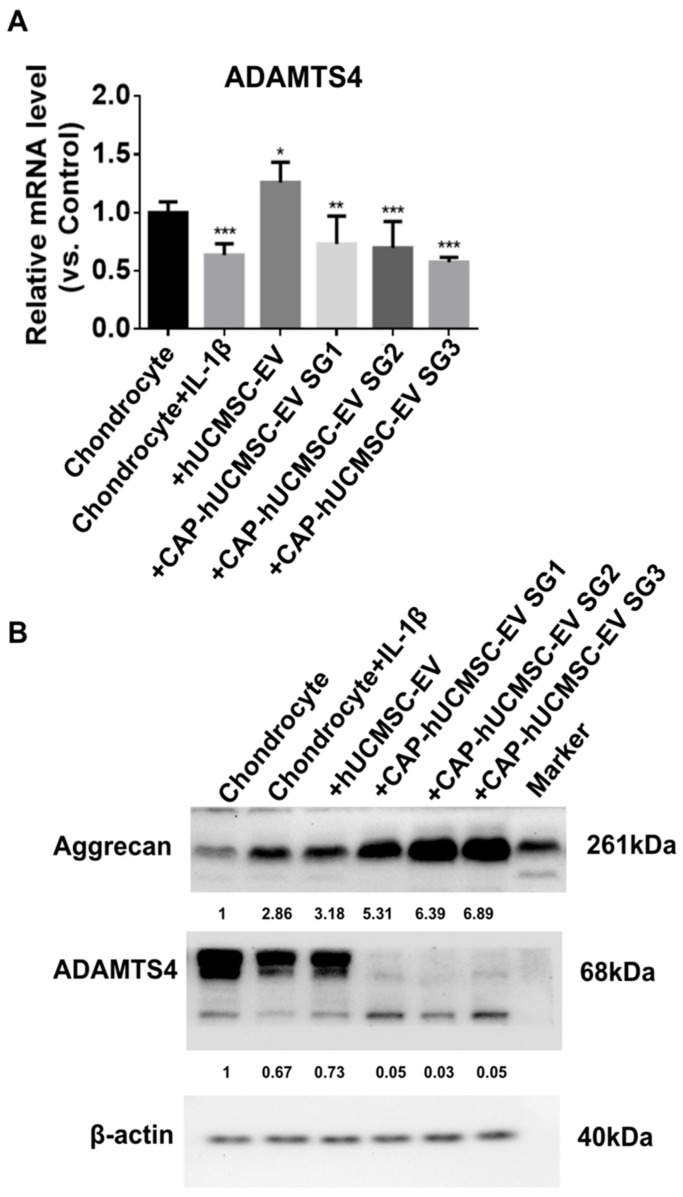
Effects of CAP-EV treatment on ADAMTS4 expression and cartilage matrix proteins in IL-1β-stimulated chondrocytes. (**A**) Quantitative real-time PCR of *ADAMTS4* mRNA levels under different treatments: untreated control, IL-1β (10 ng/mL for 48 h), hUCMSC-EV SG3, CAP-hUCMSC-EV SG1, CAP-hUCMSC-EV SG2, and CAP-hUCMSC-EV SG3 (for 24 h). Data are presented as mean ± SEM (n = 3). * *p* < 0.05, ** *p* < 0.01, *** *p* < 0.001 vs. IL-1β group. (**B**) Western blot of aggrecan (~261 kDa), ADAMTS4 (~68 kDa), and β-actin (40 kDa). Red arrowheads indicate target protein bands.

Treatment with IL-1β markedly upregulated ADAMTS4 mRNA expression in chondrocytes compared to untreated controls (Figure 5A). Administration of hUCMSC-EV SG3 further increased ADAMTS4 expression, whereas all CAP-hUCMSC-EV treatment groups (SG1, SG2, SG3) significantly suppressed *ADAMTS4* mRNA levels relative to IL-1β treatment, with the strongest inhibition observed in CAP-hUCMSC-EV SG3. Western blot analysis (Figure 5B) revealed that IL-1β stimulation decreased aggrecan protein levels and increased ADAMTS4 expression compared to the controls. CAP-hUCMSC-EV treatment restored aggrecan expression and attenuated ADAMTS4 upregulation. β-actin served as the loading control, confirming equal protein loading. These findings indicate that CAP-hUCMSC-EVs effectively counteract IL-1β-induced catabolic changes in chondrocytes.

## 3. Discussion

In this study, we isolated and characterized EVs from hUCMSCs and engineered them with CAP-LAMP2b to enhance delivery capacity and therapeutic potential. The isolated EVs exhibited a characteristic morphology, particle size, and surface marker expression (CD9, CD63, CD81, and HSP70), consistent with their exosomal identity. The expression of FLAG-tagged CAP-LAMP2b confirmed successful engineering of the vesicles, with incorporation into both cells and EVs. CAP-LAMP2b-modified EVs retained typical biophysical characteristics while offering enhanced intracellular uptake in target chondrocytes, as confirmed by DiI fluorescence imaging and quantitative uptake analysis. Notably, CAP-LAMP2b hybrid EVs significantly downregulated the matrix-degrading enzyme ADAMTS4 at both the mRNA and protein levels, while restoring aggrecan expression—a critical component of the cartilage extracellular matrix.

EVs and liposomes are both lipid bilayer-enclosed particles, but their correlation lies in their structural similarities and overlapping applications in drug delivery and cell communication research. EVs are naturally secreted by cells and carry proteins, nucleic acids, and lipids for intercellular signaling, while liposomes are synthetic vesicles engineered to encapsulate therapeutic agents [15,16,17]. Both share a phospholipid bilayer architecture, which influences their biophysical properties, cellular uptake, and ability to protect and deliver cargo. However, EVs possess complex surface proteins and lipids derived from their parent cells, conferring unique biological functions and targeting capabilities, whereas liposomes can be precisely engineered for size, composition, and surface modifications [15,16,18,19,20]. Recent research has explored hybrid systems that fuse EVs with functionalized liposomes, aiming to combine the natural targeting and low immunogenicity of EVs with the tunable properties of liposomes [21]. Liposomes are also used as models to study EV behavior and as tools to capture or mimic EVs in experimental systems [22,23]. In summary, the correlation is primarily structural and functional, as both are lipid-based vesicles with overlapping roles in drug delivery and cell communication. However, EVs are biologically derived and heterogeneous, whereas liposomes are synthetic and customizable. Advances in hybrid vesicle technology and biomimetic liposome design are leveraging these correlations to improve therapeutic delivery and biological research [15,16].

ADAMTS4 and ADAMTS5 are key enzymes responsible for aggrecan degradation in cartilage, contributing to OA progression [5,24]. These proteases play essential roles in extracellular matrix formation, homeostasis, and remodeling and drive tissue destruction in pathological conditions [25]. Inhibition strategies have evolved from zinc chelation to more selective exosite inhibition, though challenges remain in achieving isotype-specific inhibitors [5,25]. ADAMTS4 expression is synergistically upregulated by cytokines IL-1α, TNF-α, and TGF-β in OA synovial fibroblasts, primarily through TAK1, NF-κB, and ALK5/Smad2/3 signaling pathways [26]. Potential therapeutic approaches include targeting these pathways and combining anti-TNF-α drugs with other inhibitors to prevent aggrecan degradation in OA [26]. Our study further demonstrated that targeting ADAMTS4 reduced its expression while increasing aggrecan levels.

Recent advancements in CRISPR-based genome editing highlight the importance of efficient delivery systems. Engineered EVs have emerged as promising vehicles for CRISPR delivery, offering reduced immunogenicity and targeted delivery [27,28]. Researchers have developed highly potent EVs incorporating bio-inspired attributes, such as engineered mini-intein proteins for active cargo loading and fusogenic proteins for endosomal escape [29]. These EVs demonstrated high genome editing efficiency in vitro and in vivo, with potential therapeutic applications [29,30]. Strategies for improving EV-mediated CRISPR delivery include optimizing cargo loading, surface functionalization, and enhancing tissue-specific targeting [30]. While challenges remain, integrating CRISPR genome editors with engineered EVs represents a major advancement in therapeutic genome editing. In our study, a CRISPR-based genome-editing approach targeting ADAMTS4, delivered via engineered EVs (CAP-LAMP2b), effectively suppressed ADAMTS4 expression and enhanced aggrecan levels.

CRISPR/Cas9 gene editing shows promise as a disease-modifying treatment for OA. This technology can target genetic and epigenetic alterations in OA, suppressing or deleting gene expressions that drive disease [31]. CRISPR-mediated ablation of genes such as MMP13 and IL-1β reduces cartilage-degrading enzymes and attenuates structural deterioration in OA models [32]. The technology also enables the development of “designer” cells with modified receptors or gene regulatory networks, opening avenues for novel cell-based therapies [33]. Furthermore, CRISPR-based genome, epigenome, and RNA editing tools can target genetic risk factors, activate chondrogenic elements, and modulate inflammatory regulators, potentially providing personalized OA treatments [34]. Our study also applied this system for OA treatment; however, further research is needed to assess its efficacy and safety in human OA.

After hybrid formation, the particle size distribution of the CAP-LAMP2b–modified hUCMSC EVs increased significantly, which is expected due to the incorporation of liposomal CRISPR/Cas9 cargo. While larger vesicles may exhibit altered biodistribution and uptake kinetics compared with parental EVs, prior studies have shown that particles within this size range still retain efficient cellular internalization and functional delivery [35,36]. The appearance of multiple peaks in NTA suggests a heterogeneous mixture of hybrid vesicles and unfused components. Although additional purification steps (e.g., density gradient ultracentrifugation or size-exclusion chromatography) could further enrich the pure hybrid population [37], this proof-of-concept study focused on demonstrating feasibility; future optimization of the separation protocol will be important for translational applications.

These findings underscore the therapeutic potential of bioengineered EVs in modulating inflammation and preserving extracellular matrix integrity in cartilage injury or osteoarthritis. The use of CAP-LAMP2b and ADAMTS4 gene editing as targeting enhancers may improve EV retention and uptake in specific tissues, offering a platform for non-cell-based regenerative interventions. Future studies should investigate the in vivo biodistribution, safety, and functional durability of CAP-EVs, as well as their ability to deliver therapeutic RNAs or proteins in disease models.

## 4. Materials and Methods

### 4.1. Culture of hUCMSCs

The human umbilical MSCs used in this study were collected from the remaining specimens of previous studies. Thus, no additional ethical concerns were raised and approved by the Ethics Research Committee of Hualien Tzu Chi Hospital (IRB112-218-B). All procedures were conducted in a laminar flow cabinet. Wharton’s jelly was transferred to the laboratory, disinfected, and cut into 0.5–1 mm^2^ pieces. These pieces were transferred to 10 cm plates containing Low-Glucose Dulbecco’s Modified Eagle Medium (LG-DMEM, Gibco, Grand Island, NY, USA) supplemented with 10% Fetal Bovine Serum (FBS, Biological Ind., Kibbutz, Israel), 100 U/mL penicillin, 100 μg/mL streptomycin (Gibco, Waltham, MA, USA), and 2 mM L-glutamine (Gibco). The explants were rinsed in phosphate-buffered saline (PBS, Biowest, Nuaille, France) to eliminate blood clots and then incubated in a humidified atmosphere of 5% CO_2_ at 37 °C. Medium was replaced every two days, and cells migrated from the explants to the plate margins. Once cultures reached 90% confluence during the second medium change, adherent cells were harvested with 0.25% trypsin–ethylene diamine tetraacetic acid (EDTA) (Gibco). Single-cell suspensions were reseeded at a density of 5 × 10^5^ cells per 25 cm^2^ flask and used for subsequent experiments [38].

### 4.2. Flow Cytometry

hUCMSCs were incubated with fluorescent dye-conjugated antibodies for 30 min on ice. After incubation, cells were washed twice with PBS to remove unbound antibodies and incubated in blocking buffer (PBS with 2% FBS) for 10–15 min to minimize nonspecific binding. Stained cells were analyzed using a FACSCalibur flow cytometer (BD Biosciences, Franklin Lakes, NJ, USA) to assess surface marker expressions. Unstained cells served as negative controls. Antibodies included CD29, CD34, CD44, CD45, CD73, CD105, HLA-DR, and HLA-ABC (BD Biosciences).

### 4.3. Trilineage Differentiation

#### 4.3.1. Adipogenesis

For adipogenic induction, 5 × 10^4^ cells per well were seeded in 12-well plates and cultured to 60–80% confluence. Growth medium was replaced with adipogenic differentiation medium containing DMEM, 10% FBS, 5 μg/mL insulin, 1 μmol/L dexamethasone, 60 μmol/L indomethacin, and 0.5 mmol/L isobutylmethylxanthine (Sigma, St. Louis, MO, USA). Cells were maintained for 14 days with medium changes every 2–3 days. Lipid accumulation was visualized via Oil Red O staining (Sigma) and quantified via absorbance at 510 nm. All experiments were performed in triplicate.

#### 4.3.2. Osteogenesis

For osteogenic induction, 1 × 10^4^ cells per well were seeded in 12-well plates and cultured to 60–80% confluence. The medium was then replaced with osteogenic differentiation medium consisting of DMEM, 10% FBS, 50 μmol/L ascorbate, 0.1 μmol/L dexamethasone, and 10 mmol/L β-glycerol phosphate (all from Sigma). Cells were cultured for 14 days with medium changes every 2–3 days. Mineralized matrix formation was assessed via Alizarin Red S staining (Sigma) and quantified via dissolving the stain in 10% acetic acid, followed by absorbance measurement at 562 nm on an ELISA reader (Fisher Scientific, Hampton, NH, USA).

#### 4.3.3. Chondrogenesis

Chondrogenic differentiation was induced using the pellet culture method. A suspension of 2.5 × 10^7^ cells/mL was centrifuged at 1000 rpm for 5 min to form pellets, which were cultured in chondrogenic medium containing DMEM, 10% FBS, 6.25 μg/mL insulin (Sigma), 10 ng/mL transforming growth factor-β1 (PeproTech, Rocky Hill, NJ, USA), and 50 μg/mL ascorbic acid-2-phosphate (Sigma). Pellets were maintained for 21 days with medium changes every 2–3 days. Differentiation was confirmed via immunostaining for type II collagen and aggrecan.

### 4.4. Immunohistochemical Staining

Pellets were fixed in 4% paraformaldehyde for 24 h at 4 °C, embedded in OCT compound (Sigma), and cryosectioned at 10–20 μm. Sections were air-dried for 30 min, blocked with 5% normal serum and 0.1% Triton X-100 (Sigma) in PBS for 1 h, and incubated overnight at 4 °C with primary antibodies against type II collagen and aggrecan (1:200; Sigma-Aldrich, St. Louis, MO, USA). After PBS washes, sections were incubated with HRP-conjugated secondary antibodies (Sigma) for 1 h. Visualization was carried out via fluorescence microscopy (for fluorescent detection) or light microscopy following DAB substrate application. Nuclei were counterstained with hematoxylin (Sigma), and slides were mounted for analysis.

### 4.5. Quantitative Real-Time PCR

Total RNA was extracted using the PureLink RNA Mini Kit (Life Technologies, Carlsbad, CA, USA), and 1 μg of RNA was reverse-transcribed into cDNA using SuperScript III (Invitrogen, Waltham, MA, USA). qRT-PCR was performed with Fast SYBR Green Master Mix (Applied Biosystems, Foster City, CA, USA) on a QuantStudio 5 system (Applied Biosystems). GAPDH served as the internal control. Primers targeting PPARγ and FABP4 (adipogenesis), ALPL and RUNX2 (osteogenesis), and aggrecan and COL2A1 (chondrogenesis) were used. Negative controls included reactions lacking reverse transcription or template. Specificity was confirmed via melting curve analysis and agarose gel electrophoresis. Ct values were analyzed using the 2^−ΔΔCt^ method, normalized to GAPDH. Primer sequences are provided in Table 1.

### 4.6. CAP-Lamp2b Plasmid Development and Transfection

**Figure 6:**
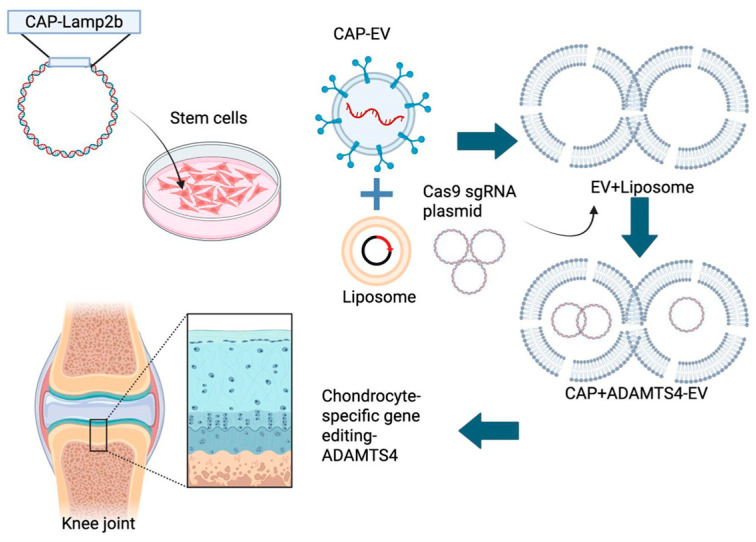
Schematic representation of the hybrid CAP-LAMP2b–modified hUCMSC extracellular vesicle (EV) delivery system. The diagram illustrates the stepwise generation of hybrid EVs for targeted chondrocyte gene editing. CAP-LAMP2b is expressed in hUCMSCs to produce CAP-engineered EV (CAP-EV). These EVs are fused with liposomes containing Cas9/sgRNA plasmids directed against ADAMTS4 to form hybrid EVs. The hybrid vesicles combine the biocompatibility of EVs and natural tropism with the engineered CAP-LAMP2b peptide to enhance chondrocyte targeting, enabling the efficient delivery of CRISPR–Cas9 cargo and chondrocyte-specific ADAMTS4 gene editing to counteract IL-1β–induced aggrecan loss.

Flag-Lamp2b-HA and CAP (chondrocyte affinity peptide)-Flag-Lamp2b-HA DNA plasmids were designed and constructed by BIOTOOLS Co., Ltd. (New Taipei City, Taiwan). The CAP-LAMP2B sequence (1281 bp, Clone ID: TK275677) was cloned into the pcDNA3.1(+)-N-DYK vector using the KpnI/BamHI restriction sites. The ligated plasmid was transformed into competent E. coli, and positive clones were confirmed via sequencing. These plasmids were transfected into hUCMSCs for stable retention and expression. For transfection, Lipofectamine 3000 reagent (Thermo Fisher, Waltham, MA, USA) was prepared according to the manufacturer’s instructions. Briefly, 7.5 µL Lipofectamine 3000 reagent and 5 µg nucleic acids (plasmid DNA) were separately diluted in Opti-MEM medium (125 µL and 250 µL, respectively, Thermo Fisher). The diluted Lipofectamine 3000 reagent and nucleic acids were then gently mixed at a 1:1 ratio (125 µL: 125 µL) and incubated for 20 min to allow formation of transfection complexes. Following 24 h transfection, the cells were subjected to antibiotic selection. The ideal concentration of Hygromycin B (Sigma-Aldrich) for selecting and maintaining CAP-Lamp2b-transfected cell lines was determined via an Antibiotic Kill Curve Test. The optimal dose was defined as the lowest concentration at which all cells were eliminated after seven days of treatment. With resistance conferred by the Flag-Lamp2b-HA and CAP-Flag-Lamp2b-HA plasmids, successfully transfected hUCMSCs survived selection under a high dose of Hygromycin B. The selected hUCMSCs were cultured with Hygromycin B at the optimal dose for one week prior to EV extraction and were subsequently maintained with low doses to ensure continued plasmid expression (Figure 6).

### 4.7. EV Isolation

EVs were isolated from the conditioned medium (CM) of hUCMSCs with or without transfections. When cells reached 50–60% confluence, they were washed with PBS and incubated in serum-free defined medium (MesenGRo hMSC medium; StemRD, Burlingame, CA, USA) for 48 h. The CM was collected and centrifuged at 300× *g* for 15 min, followed by 2500× *g* for 15 min to remove cell debris and dead cells. The resulting supernatant was filtered through a 0.22 μm filter (Merck–Millipore, Burlington, MA, USA) to eliminate residual debris. For EV precipitation, 63 μL of ExoQuick solution (EXOTC50A-1; System Biosciences, Palo Alto, CA, USA) was added to 250 μL of CM, mixed, and incubated at 4 °C for at least 1 h. The mixture was centrifuged at 2000× *g* for 30 min, and the supernatant was discarded. The EV pellet was further centrifuged at 1500× *g* for 5 min to remove residual ExoQuick reagent. All isolation steps were performed under sterile conditions. The purified EVs were resuspended in EV-Guard storage buffer (EXSBA-1; System Biosciences) and stored at −80 °C until use.

### 4.8. Western Blot

Protein expression of Flag, CD9, CD63, and CD81 was analyzed to characterize exosomes derived from transfected hUCMSCs. The Flag tag marker was incorporated into targeted CAP-Flag-Lamp2b and non-targeted Flag-Lamp2b constructs to confirm transgene expression. Western blot analysis was performed to evaluate the expression of CD9, CD63, CD81, and HSP70. EVs were lysed with protein lysis buffer (Sigma-Aldrich) to extract total proteins, which were separated by 10% sodium dodecyl sulfate–polyacrylamide gel electrophoresis (SDS-PAGE; Sigma-Aldrich) and transferred to membranes. Membranes were incubated overnight at 4 °C with primary antibodies against CD9, CD63, CD81, and HSP70 (1:2000; Sigma-Aldrich), followed by incubation with horseradish peroxidase (HRP)-conjugated secondary antibodies (1:5000; Sigma-Aldrich). Protein bands were visualized using an electrochemiluminescence detection kit (Promega, Fitchburg, WI, USA). For in vitro experiments, chondrocytes were stimulated with IL-1β (10 ng/mL) for 24 h and then cultured with or without hybrid EVs. Proteins from each group were extracted, and expression levels of ADAMTS4 and aggrecan (Cell Signaling, Danvers, MA, USA) were analyzed via Western blotting. Western blotting was also performed using anti-DDDDK (FLAG, Abcam, Cambridge, England, UK).

### 4.9. Transmission Electron Microscope

Isolated EVs were fixed with 2.5% glutaraldehyde in cacodylate buffer and incubated at room temperature for 2 h. Fixed EVs were washed with cacodylate buffer (Sigma-Aldrich) and post-fixed with 1% osmium tetroxide (Sigma-Aldrich) for 1 h. Samples were dehydrated in a graded ethanol series and embedded in epoxy resin (Sigma-Aldrich). Ultrathin sections were cut using an ultramicrotome and mounted on copper grids (697745, Sigma). Grids were stained with uranyl acetate and lead citrate to enhance contrast. EVs were then examined under a transmission electron microscope (TEM) at appropriate magnifications, and images were acquired for analysis (HITACHI H-7500, Tokyo, Japan). TEM images provided insights into the structural features of EVs, including size, shape, and characteristic lipid bilayers.

### 4.10. Nanoparticle Tracking Analysis

Isolated EVs were diluted in a suitable buffer to achieve an optimal particle concentration for analysis. The diluted EV samples were loaded into the sample chamber of a Nano-Sight NS300 instrument (Malvern, Worcestershire, UK), and laser light scattering was used to detect and track the Brownian motion of individual EVs. The acquired data were analyzed using specialized software to calculate the mean size, size distribution, and concentration of the EVs. The NTA provided quantitative information on the size range of exosomes, enabling the detection of subpopulations within the sample. Additionally, the concentration measurement offered insights into the yield and abundance of EVs (particles/mL).

### 4.11. CRISPR Cas9 Plasmid Construction and Transfection

Three CRISPR Cas9 gRNAs were designed and constructed by GeneDireX Inc. (Taoyuan, Taiwan). The following protocol was used to transfect CRISPR Cas9 plasmids expressing gRNA for ADAMTS4 into EVs for efficient delivery into chondrocytes. For targeted knockout of ADAMTS4 (homo sapiens), the designed gRNA sequences against ADAMTS4 were cloned under the U6 promoter into the pSpCas9 BB-2A-Puro (PX459) v2.0 plasmid (Addgene, Watertown, MA, USA). This plasmid co-expresses SpCas9 and the ADAMTS4-specific guide RNA (gRNA), enabling site-specific DNA cleavage. The construct also carries a puromycin resistance gene for selection and a CMV enhancer/chicken β-actin promoter to ensure robust expression of Cas9 in mammalian cells. Following transfection, Cas9 introduces double-strand breaks at the ADAMTS4 locus, which are subsequently repaired via non-homologous end joining, resulting in gene disruption. Hybrid EVs loaded with the CRISPR-Cas9 plasmid were then applied to IL-1β-treated chondrocytes to evaluate the effect on ADAMTS4 expression and aggrecan preservation. The sgRNA sequences targeting ADAMTS4 are provided in the Supplementary Information (Figure S1). EVs were isolated from cell culture supernatants. For transfection, the Lipofectamine 3000 reagent (Thermo Fisher) was prepared according to the manufacturer’s instructions. Briefly, 7.5 µL Lipofectamine 3000 reagent was diluted in 125 µL Opti-MEM medium (Thermo Fisher). Then, 5 µg plasmid DNA encoding Cas9 and ADAMTS4 gRNA were diluted in 250 µL Opti-MEM medium. The diluted Lipofectamine 3000 reagent and plasmid were then mixed at a 1:1 ratio (125 µL: 125 µL) gently and incubated for 20 min to allow the formation of transfection complexes. The transfection complexes were added to the isolated EVs and gently mixed, followed by incubation at room temperature for 30–60 min. As Cas9 plasmid-carrying hybrid EVs reached the IL-1β-treated chondrocytes (treated for 48 h), the hybrid EVs were expected to be engulfed by the surrounding cells, leading to Cas9 plasmid expression for targeted genome editing [39].

### 4.12. Human Chondrocyte Culture

Human chondrocytes were isolated from cartilage samples obtained from human donors. The cartilage was minced into small fragments and digested overnight with type II collagenase diluted in culture medium. The digestion mixture was then passed through a 70 μm cell strainer (Falcon, BD Biosciences, Franklin Lakes, NJ, USA) to remove debris. The resulting cell suspension was seeded into 10 cm culture dishes containing 10 mL of DMEM/F12 medium (Sigma) supplemented with 10% FBS and 1% penicillin/streptomycin. Chondrocytes were maintained for 14 days before passaging.

### 4.13. CAP Hybrid EV Retention Measurement in Chondrocytes

To further assess the uptake of EVs by chondrocytes, CAP and non-CAP hybrid EVs were labeled with DiI (Thermo Fisher), a lipophilic carbocyanine dye that selectively labels membrane-bound vesicles. Fluorescent labeling of the CAP hybrid EVs with DiI enabled confocal microscopy analysis, which revealed distinct fluorescent signals within the cytoplasm of chondrocytes, indicating the successful internalization of the hybrid vesicles. This observation provides evidence for the efficient cellular uptake of DiI-labeled CAP hybrid exosomes by primary chondrocytes. To assess the cellular internalization of CAP hybrid exosomes in primary chondrocytes, an equal volume of DiI-labeled CAP or non-CAP hybrid exosomes was applied to the cells in three independent experiments, while control wells received only DMEM. The cells were incubated with the DiI-tagged exosomes for two hours, and images were captured using Zeiss ZEN software (version 3.6, Oberkochen, Germany). Confocal microscopy, comparing control, CAP, and non-CAP hybrid EVs, allowed for the tracking and comparison of cellular uptake and intracellular movement of synthetic nanocarriers at the cellular level.

### 4.14. Induction of OA in Chondrocytes

To simulate the pathophysiology of OA in chondrocytes, interleukin-1 beta (IL-1β) treatment was employed, as it is widely recognized for inducing OA-like symptoms [40]. Specifically, the aim was to elevate the expression of matrix aggrecanase-1 (ADAMTS4) in chondrocytes by subjecting them to IL-1β stimulation. By replicating the pathological conditions associated with OA, this experimental approach enabled investigation of the impact of increased ADAMTS4 expression in chondrocytes, providing insights into the molecular mechanisms underlying OA progression. Cells were seeded into 6-well plates (2–3 × 10^5^ cells/well for gene/protein analysis) and cultured overnight to ~70% confluence. The next day, cells were washed with PBS and incubated in serum-free or 1% FBS medium containing recombinant human IL-1β (10 ng/mL; PeproTech, USA) for 24 or 48 h, with untreated cells serving as controls. Cells were harvested for qRT-PCR and Western blot analysis of inflammation-related genes (*ADAMTS4*) and chondrocyte protein (aggrecan, Cell Signaling). All experiments were performed in triplicate.

### 4.15. Statistical Analysis

Data are expressed as means ± standard error of the mean (SEM). qRT-PCR data were analyzed using one-way repeated-measures ANOVA or one-way ANOVA, followed by Fisher’s least significant difference (LSD) post hoc test. A *p*-value < 0.05 was considered statistically significant.

## 5. Conclusions

In this study, we demonstrated that CAP-LAMP2b-engineered EVs derived from hUCMSCs can efficiently deliver CRISPR-Cas9 plasmids targeting ADAMTS4. These engineered EVs successfully reduced ADAMTS4 expression and preserved aggrecan levels in IL-1β-treated chondrocytes, thereby mitigating extracellular matrix degradation under inflammatory conditions. Our findings provide proof of concept that gene-modified EVs represent a promising therapeutic platform for cartilage repair. Based on these results, we recommend that future research should focus on validating the efficacy and safety of this strategy using in vivo OA models, optimizing delivery efficiency, and exploring the translational potential of engineered EVs in regenerative medicine.

## Figures and Tables

**Table 1:** Primer sequence of candidate genes.

Gene Name	Forward Sequence (5′-3′)	Reverse Sequence (5′-3′)	Product Size (bp)
*PPARγ*	AGCCTCATGAAGAGCCTTCCA	TCCGGAAGAAACCCTTGCA	120
*FABP4*	ATGGGATGGAAAATCAACCA	GTGGAAGTGACGCCTTTCAT	87
*ALPL*	CCACGTCTTCACATTTGGTG	GCAGTGAAGGGCTTCTTGTC	96
*RUNX2*	CGGAATGCCTCTGCTGTTAT	TTCCCGAGGTCCATCTACTG	174
*Aggrecan*	GAGATGGAG GGTGAGGTC	ACGCTGCCTCGGGCTTC	443
*COL2A1*	GGACTTTTCTCCCCTCTCT	GACCCGAAGGTCTTACAGGA	104
*ADAMTS4*	TCACTGACTTCCTGGACAATGGC	GGTCAGCATCATAGTCCTTGCC	105
*GAPDH*	GAAGGTGAAGGTCGGAGTC	GAAGA TGGTGATGGGATTTC	172

## Data Availability

The raw data supporting the conclusions of this article will be made available by the authors on request.

## Supplementary Materials

The following supporting information can be downloaded at: https://www.mdpi.com/article/10.3390/ijms26199812/s1.

## Abbreviations

The following abbreviations are used in this manuscript:

OA	Osteoarthritis
hUCMSC	Human umbilical cord MSC
EV	Extracellular vesicle
MMP	Matrix metalloproteinase
