# Supplementary material for: CAP-LAMP2b–Modified Stem Cells’ Extracellular Vesicles Hybrid with CRISPR-Cas9 Targeting ADAMTS4 to Reverse IL-1β–Induced Aggrecan Loss in Chondrocytes
Source: Int J Mol Sci. 2025 Oct 9;26(19):9812. doi: 10.3390/ijms26199812 (PMC12525490; doi:10.3390/ijms26199812)
Supplement: Supplementary file 1 [file ijms-26-09812-s001.zip › ijms-3862746-supplementary.pdf]

Figure S1. Cas9 sgRNAs sequence

Guide #1: CAGCCCCUCGACCUGCACAC

Download GenBank Graphics ▼ Next ▲ Previous ◀ Descriptions

Homo sapiens ADAM metallopeptidase with thrombospondin type 1 motif 4 (ADAMTS4), transcript variant 1, mRNA

Sequence ID: NM_005099.6 Length: 9777 Number of Matches: 1

Range 1: 729 to 748 GenBank Graphics ▼ Next Match ▲ Previous Match

Score	Expect	Identities	Gaps	Strand
40.1 bits(20)	1.4	20/20(100%)	0/20(0%)	Plus/Minus

```
Query  1    CAGCCCCTCGACCTGCACAC  20
            ||||||||||||||||||||
Sbjct  748  CAGCCCCTCGACCTGCACAC  729
```

Related Information
Gene - associated gene details
PubChem BioAssay - bioactivity screening
Genome Data Viewer - aligned genomic context

Guide #2: CAGGCUGUUGUGCCGCUUGC

Download GenBank Graphics ▼ Next ▲ Previous ◀ Descriptions

Homo sapiens ADAM metallopeptidase with thrombospondin type 1 motif 4 (ADAMTS4), transcript variant 1, mRNA

Sequence ID: NM_005099.6 Length: 9777 Number of Matches: 1

Range 1: 664 to 683 GenBank Graphics ▼ Next Match ▲ Previous Match

Score	Expect	Identities	Gaps	Strand
40.1 bits(20)	1.4	20/20(100%)	0/20(0%)	Plus/Plus

```
Query  1    CAGGCTGTTGTGCCGCTTGC  20
            ||||||||||||||||||||
Sbjct  664  CAGGCTGTTGTGCCGCTTGC  683
```

Related Information
Gene - associated gene details
PubChem BioAssay - bioactivity screening
Genome Data Viewer - aligned genomic context

Guide #3: AAACACGAUCUCCUCCUCCC

Download GenBank Graphics ▼ Next ▲ Previous ◀ Descriptions

Homo sapiens ADAM metallopeptidase with thrombospondin type 1 motif 4 (ADAMTS4), transcript variant 2, mRNA

Sequence ID: NM_001320336.3 Length: 4170 Number of Matches: 1

Range 1: 597 to 616 GenBank Graphics ▼ Next Match ▲ Previous Match

Score	Expect	Identities	Gaps	Strand
40.1 bits(20)	1.4	20/20(100%)	0/20(0%)	Plus/Minus

```
Query  1    AAACACGATCTCCTCCTCCC  20
            ||||||||||||||||||||
Sbjct  616  AAACACGATCTCCTCCTCCC  597
```

Related Information
Gene - associated gene details
PubChem BioAssay - bioactivity screening
Genome Data Viewer - aligned genomic context

Figure 2

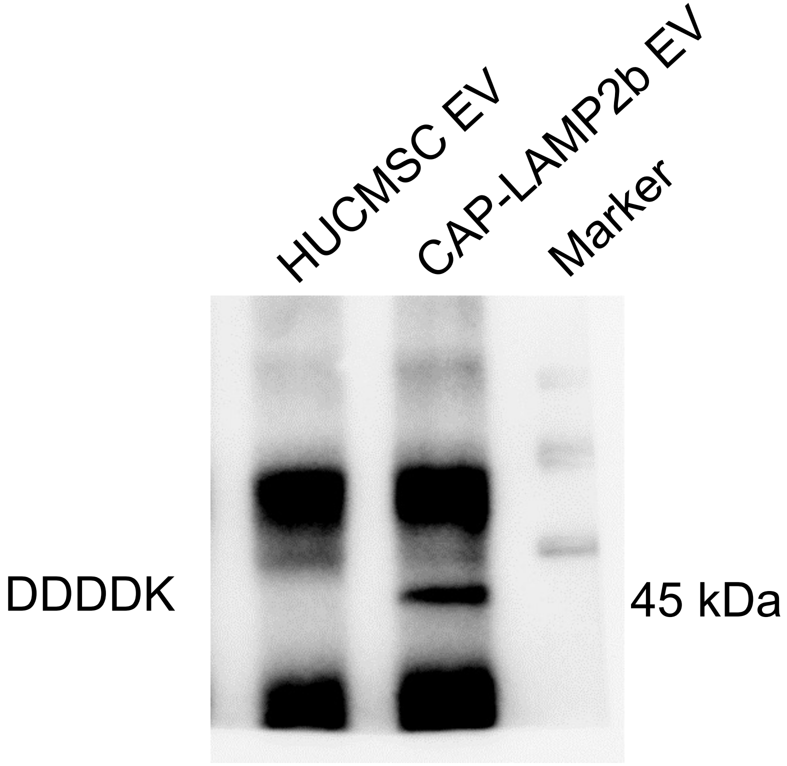

Figure 3G

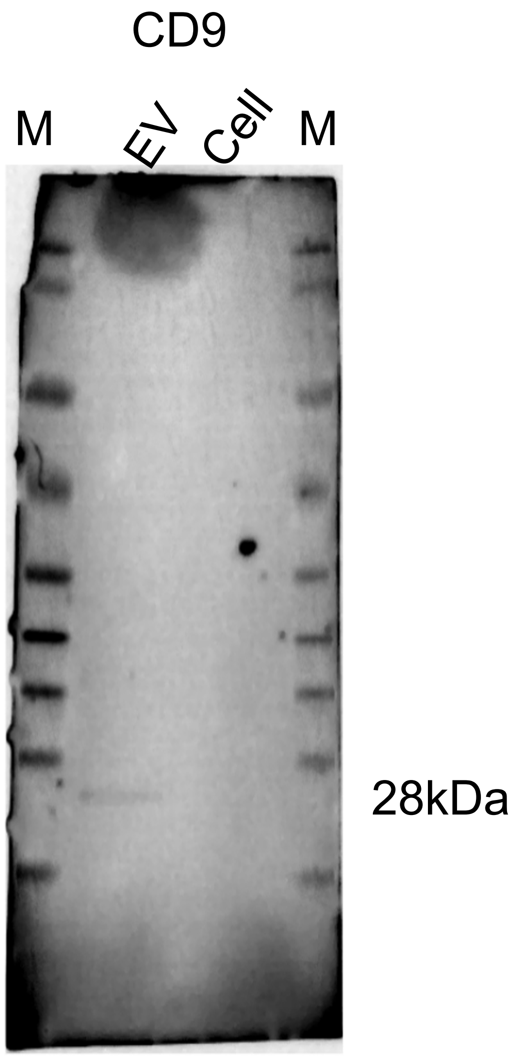

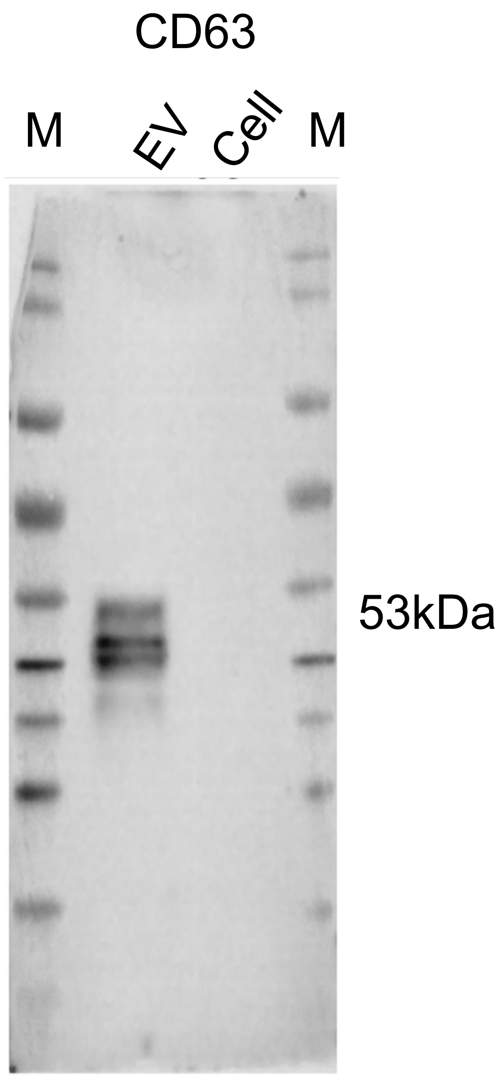
CD63
M
EV
Cell
M
53kDa

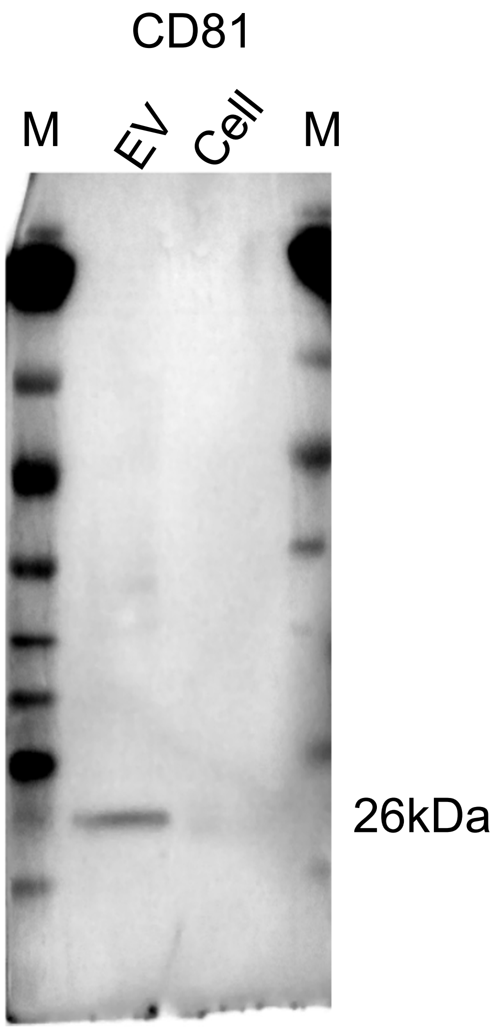
CD81
M
EV
Cell
M
26kDa

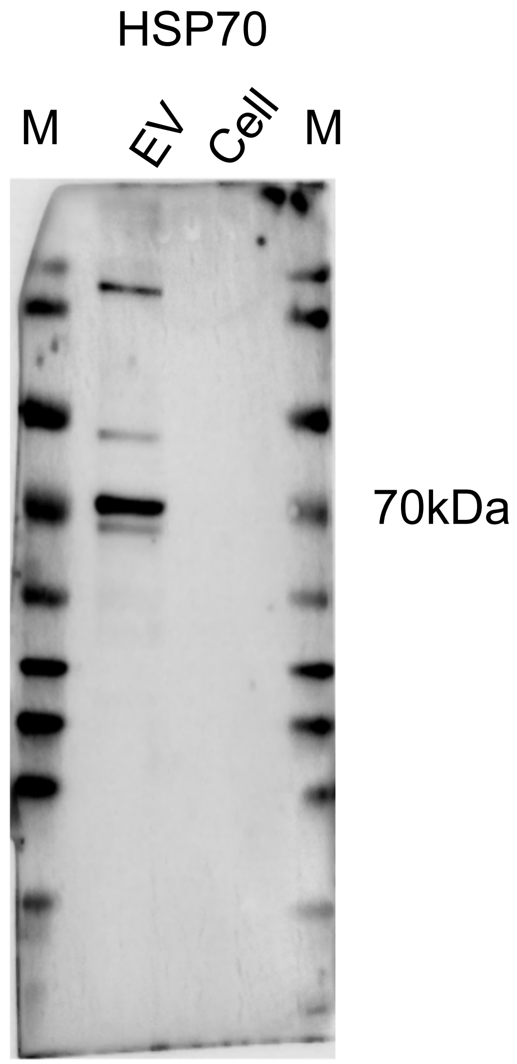
HSP70
M
EV
Cell
M
70kDa

Figure 5B

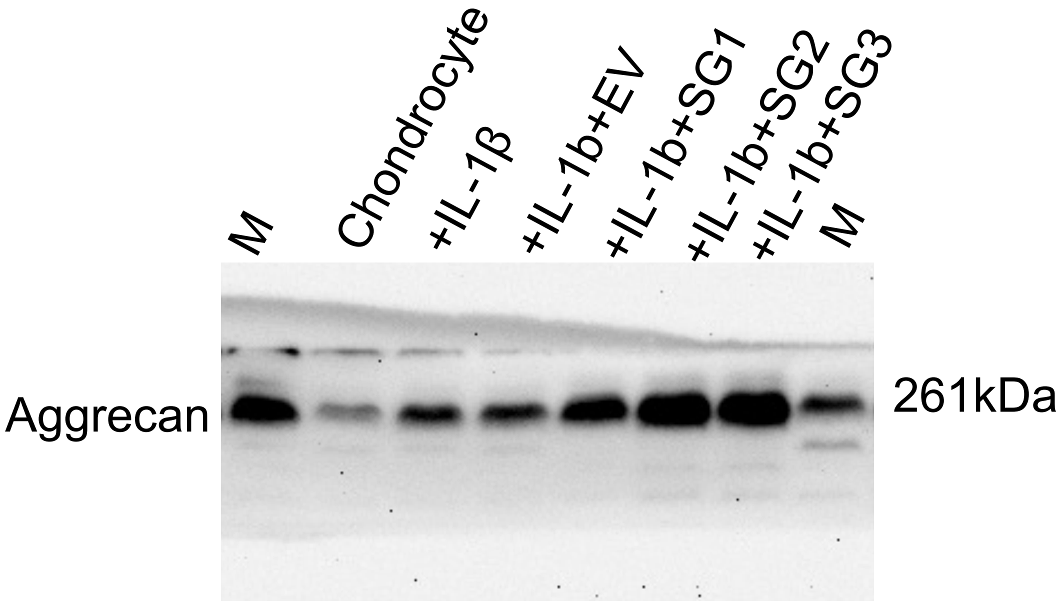
M
Chondrocyte
+IL-1β
+IL-1b+EV
+IL-1b+SG1
+IL-1b+SG2
+IL-1b+SG3
M
Aggrecan
261kDa

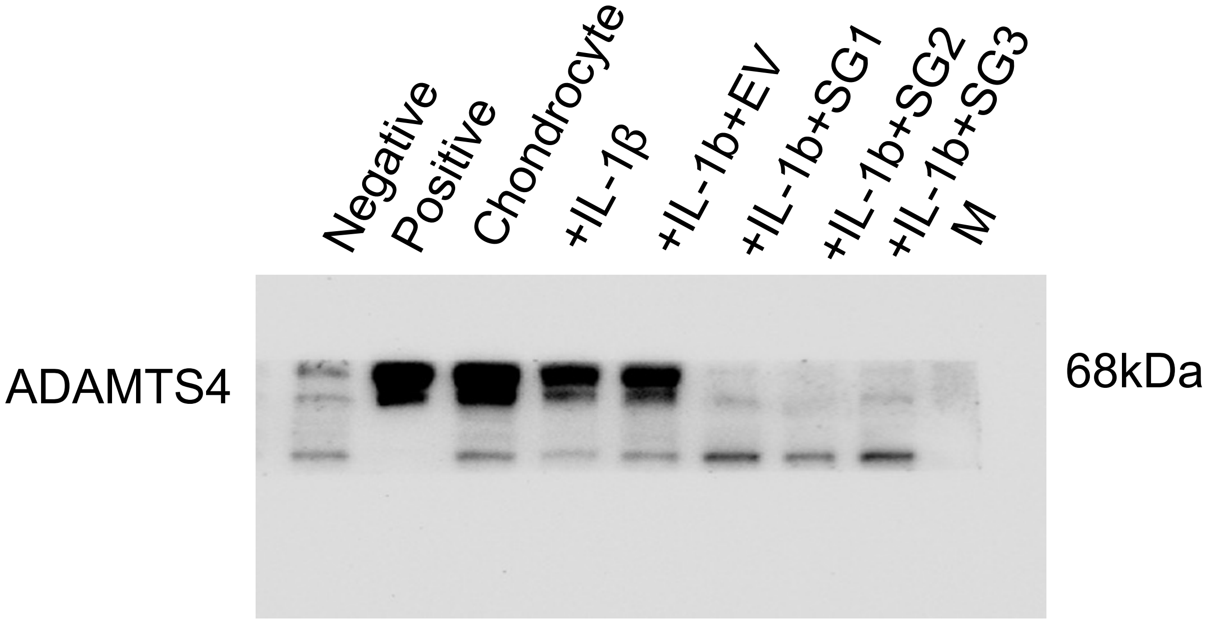
Negative
Positive
Chondrocyte
+IL-1β
+IL-1b+EV
+IL-1b+SG1
+IL-1b+SG2
+IL-1b+SG3
M
ADAMTS4
68kDa

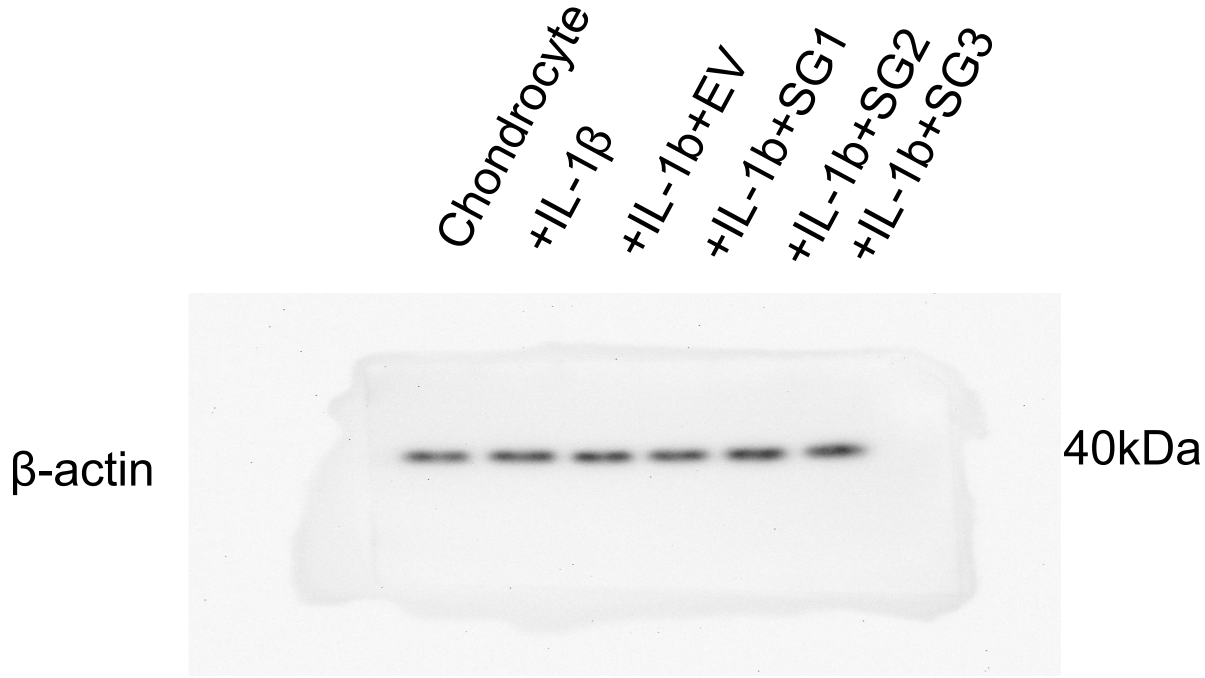
Chondrocyte
+IL-1β
+IL-1b+EV
+IL-1b+SG1
+IL-1b+SG2
+IL-1b+SG3
β-actin
40kDa
